# Biodistribution of Polyaldehydedextran Nanoparticle-Encapsulated Epirubicin in Ovarian Tumor-Bearing Mice via Optical Imaging

**DOI:** 10.3390/ijms26030970

**Published:** 2025-01-24

**Authors:** Wioletta Kośnik, Hanna Sikorska, Adam Kiciak, Tomasz Ciach

**Affiliations:** 1 NanoVelos S.A., Rakowiecka 36, 02-532 Warsaw, Poland; 2 NanoGroup S.A., Rakowiecka 36, 02-532 Warsaw, Poland; 3 Faculty of Chemical and Process Engineering, Warsaw University of Technology, Waryńskiego 1, 00-645 Warsaw, Poland

**Keywords:** polysaccharide-based nanoparticles, epirubicin, biodistribution, optical imaging, ovarian cancer, patient-derived xenograft (PDX)

## Abstract

This study investigates the biodistribution of polysaccharide-based nanoparticles loaded with epirubicin (POLEPI) compared to epirubicin hydrochloride (EPI) in naïve female nude mice following a single intravenous dose. The inherent fluorescence of epirubicin was tracked using Newton 7 animal imager and Varioskan. Initial whole-animal optical imaging failed to reliably detect epirubicin distribution, necessitating ex vivo imaging of key tissues harvested at intervals between 10 min and 48 h post-injection. Optimal imaging conditions were established using a 5 s exposure time with excitation (Ex)/emission (Em) at 480 nm/550 nm. The biodistribution of POLEPI was further evaluated in both naïve mice and immunocompromised mice bearing patient-derived ovarian tumors. Unlike epirubicin, POLEPI exhibited notable tissue distribution within 3 h post-injection. By 48 h, fluorescence signals were undetectable in both models, although non-tumored animals exhibited persistent signals. In both models, the liver was the primary organ for POLEPI accumulation, with lower levels in tumored mice. Interestingly, brain fluorescence was higher in POLEPI-treated mice compared to those receiving epirubicin. Neither POLEPI nor epirubicin accumulated in the spleen or bone marrow. In tumors, POLEPI fluorescence peaked at 24 h, with levels 2.1 times higher than in the epirubicin-treated group over a 48 h period. Furthermore, POLEPI uptake in tumors exceeded that in healthy ovaries, with the most significant tumor-to-healthy-ovary ratio observed between 6 and 24 h post-injection. These findings demonstrate that POLEPI, a novel polyaldehydedextran nanoparticle formulation, exhibits enhanced accumulation and retention in tumor tissue compared to epirubicin, with preferential distribution to the orthotopic tumor-bearing ovary over healthy ovarian tissue. The inherent fluorescence of epirubicin provided a rapid and cost-effective means of estimating biodistribution, although the limitations of this method—particularly, the inability to differentiate between the parent drug and its metabolites—were acknowledged.

## 1. Introduction

Nanoparticles (NPs) (1–100 nm) have great potential as cancer theragnostics (i.e., therapeutics and diagnostics). NPs, in combination with numerous imaging techniques (like positron emission tomography PET, single-photon emission computed tomography SPECT, magnetic resonance imaging MRI, etc.) and various advanced cancer therapeutics (like magnetic hyperthermia, pH responsiveness, photothermal therapy, etc.) have been stated to be more targeted and effective therapeutic strategies with negligible side effects in women’s cancer treatment [1]. Nanoparticles are thought to offer several benefits, such as enhancing the permeability of hydrophilic drugs, prolonging their half-life in plasma, enabling targeted delivery, and increasing the therapeutic index. Despite substantial investments and promising outcomes in preclinical studies, the translation of nanoparticles from bench to bedside has been relatively limited, with fewer than 60 clinically viable formulations based on nanoparticles [2]. But with the development and successful application of mRNA-lipid nanoparticle COVID-19 vaccines, unprecedented attention has been paid to the bench-to-bedside translation of nanomedicines worldwide [3]. A complete, as of 2 October 2023, list of cancer nano-therapeutics approved by the FDA and regulatory agencies of other countries is given on the National Cancer Institute site [4]. A total of 114 clinical trials are ongoing [5]. A crucial factor influencing the efficacy and toxicity of nanoparticles is their biodistribution profile and exposure at the action site [6] While numerous studies have explored the preclinical biodistribution of nanoparticles using various techniques, the majority have focused on determining the pharmacokinetics (PK) of drugs loaded within nanoparticles. In our research, we investigated the biodistribution of dextran nanoparticles (NPs) loaded with epirubicin through ex vivo optical imaging of dissected organs, utilizing the intrinsic fluorescence of epirubicin. Fluorescence imaging, although limited in distinguishing between the parent drug and its metabolites, offers a practical and efficient approach for initial biodistribution assessments. The quantification of extracted epirubicin in plasma was performed using HPLC/MS/MS. As previously outlined, dextran nanoparticles (NPs) were synthesized from 70 kDa dextran, with a 5% degree of oxidation of the polysaccharide chain and 50% substitution with dodecylamine, resulting in an NP backbone composed of modified dextran subunits. In an aqueous environment, these NPs exhibit a mean diameter of approximately 100 nm [7]. Notably, these dextran NPs can be stored in a dry state and reassembled in water. Additionally, it was determined that various chemical moieties, such as drugs like doxorubicin, could be linked to dextran NPs through a pH-dependent bond, facilitating drug release under lower pH conditions. The fluorescence signal of doxorubicin (Dox) delivered to cells via Dox-NPs was observed in both the cell nuclei and cytoplasm. The cytoplasmic signal originated from doxorubicin bound to the NPs, representing the unreleased fraction. To confirm whether the nuclear signal resulted from doxorubicin already released from the NPs (indicating that Dox-NPs did not enter the cell nuclei), Wasiak et al. chemically reduced the bond between doxorubicin and the NPs to prevent doxorubicin release. Consequently, the signal from reduced Dox-NPs was not detectable within the cell nuclei, confirming that the nuclear signal in the case of Dox-NPs originated from the released doxorubicin.

POLEPI refers to epirubicin encapsulated within nanoparticles. The final product comprises EPI as the active substance, polyaldehyde-dextran (PAD) serving as the backbone, dodecylamine hydrochloride (DDA) as the coiling agent, and alanine, all acting as agents in the formation of nanoparticles (Supporting Information Tables S1–S4, synthesis procedure). In a neutral pH environment, such as the bloodstream, POLEPI effectively encapsulates the drug within spherical nanoparticles, ensuring stable drug loading. However, in an acidic environment, the loaded drug is selectively released through the dissociation of the imine bond (Supporting Information Figures S1 and S2).

Ovarian cancer stands as a highly lethal gynecological malignant neoplasm characterized by a limited number of identified modifiable risk factors [8]. While platinum-based chemotherapy remains the treatment mainstay for advanced ovarian cancer, most patients develop resistance and experience relapse within the first two years of treatment [9]. Epirubicin, an aging anthracycline drug with anticancer properties, is commonly administered for treating solid cancers, including ovarian cancer. However, the accumulation of epirubicin in the human body poses a significant risk of toxicity, leading to severe adverse effects such as cardiotoxicity, myelosuppression, and hypoalbuminemia. These effects have the potential to cause irreversible damage to vital organs.

Consequently, the targeted delivery of epirubicin to tumors through nanoparticles, allowing for slow and controlled drug release, particularly within the acidic environments present in tumors, holds great appeal. To examine this hypothesis, we conducted a study tracking the biodistribution of POLEPI in immunocompromised mice, including both naïve subjects and those with orthotopic ovarian cancer patient-derived xenografts (PDX). The investigation involved optical imaging of epirubicin’s inherent fluorescence in whole animals and resected tissues, utilizing the Newton 7.0 animal imager (Vilber, Collégien, France) and Varioskan^®^ Lux (Thermo Fisher, Waltham, MA, USA, Model #N7-00020).

## 2. Results

### 2.1. Epirubicin Whole-Body Fluorescence Imagining: Subpart A1

**Figure 1:**
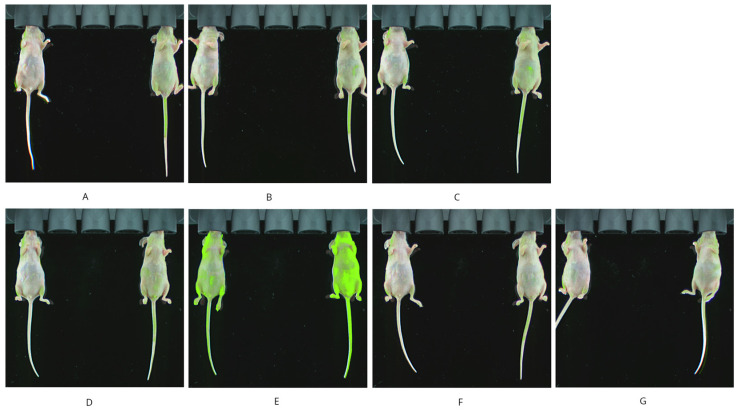
Whole-body imaging at various time intervals: 10 min (**A**), 30 min (**B**), 1 h (**C**), 3 h (**D**), 6 h (**E**), 24 h (**F**), and 48 h (**G**). Mouse on the left was injected with saline and mouse on the right with epirubicin.

The primary objective of this subsection was to acquire quantifiable images with spatial resolution to depict the distribution of epirubicin. The potential for detecting epirubicin fluorescence at various time intervals (10 min, 30 min, 1 h, 3 h, 6 h, 24 h, and 48 h) following a single intravenous dose of epirubicin was evaluated and compared to a saline vehicle at *n* = 1/group to ascertain the peak signal timepoint. Whole-body imaging was employed to assess epirubicin distribution. An average signal-to-noise ratio of 1.57 was achieved across the seven time points. However, the reliable detection of epirubicin distribution throughout the whole body was not consistently achieved, as areas of high intensity exhibited variability between preliminary imaging sessions (Figure 1).

**Figure 2:**
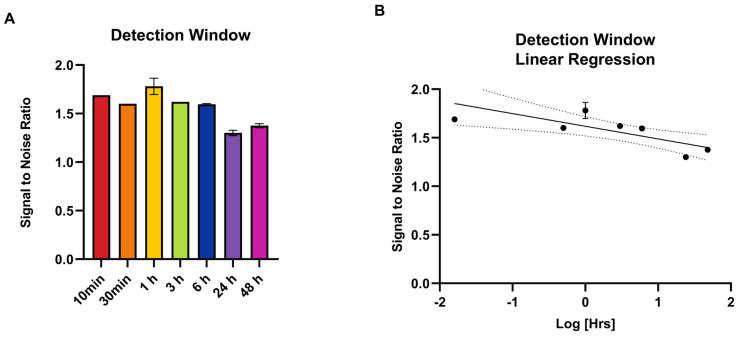
Detection window by time point (**A**) and linear regression (**B**).

The time points from 10 min to 3 h demonstrated a high signal plateau, with an optimal signal-to-noise ratio observed at 1 h (1.78) (Figure 2A), followed by logarithmic decay and subsequent signal drop-off after 6 h (Figure 2B).

Peak fluorescence was identified at Ex 580 nm/Em 600 nm; however, achieving unsaturated exposure proved challenging. The best acquisition parameters were determined to be at Ex 540 nm/Em 600 nm and a 1 s exposure. The fluorescence signal observed in our imaging approach indeed reflects a combination of the unchanged drug (epirubicin) and its fluorescent metabolites. This overlap is an inherent limitation of using fluorescence imaging for biodistribution studies, as epirubicin and its metabolites share the same fluorescence emission wavelength.

### 2.2. Epirubicin Ex Vivo Fluorescence Imaging in Resected Organs and Blood: Subpart A2

**Figure 3:**
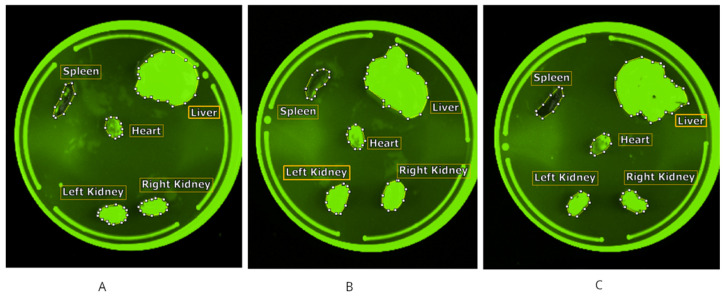
Tissue images Ex 480 nm/Em 550 nm: empty nanoparticle carrier (**A**), epirubicin (**B**), and POLEPI (**C**).

Subpart A2 aimed to assess the feasibility of detecting epirubicin and POLEPI fluorescence in organs and blood through ex vivo imaging. One mouse per group was intravenously injected with empty NP (vehicle), epirubicin, or POLEPI. From 1 to 3 h post-dose (the plateau identified in Subpart A1), each mouse was euthanized, and organs and blood were collected for imaging. Seven excitation/emission channel pairs were evaluated for signal strength and compared with the container background and empty NP control. An auto-scan feature that was exposed for up to 5 s, reaching saturation, was utilized to determine the strength of the epirubicin or POLEPI signal relative to the background signal. A 5 s exposure time and harvesting workflow were piloted for Subpart B. The background signal from the tissues was found to be tissue type-dependent: The spleen showed no signal in any treatment group, while a high background signal was observed in the liver and kidneys (Figure 3). The heart exhibited an intermediate background signal distinguishable from the epirubicin signal.

**Figure 4:**
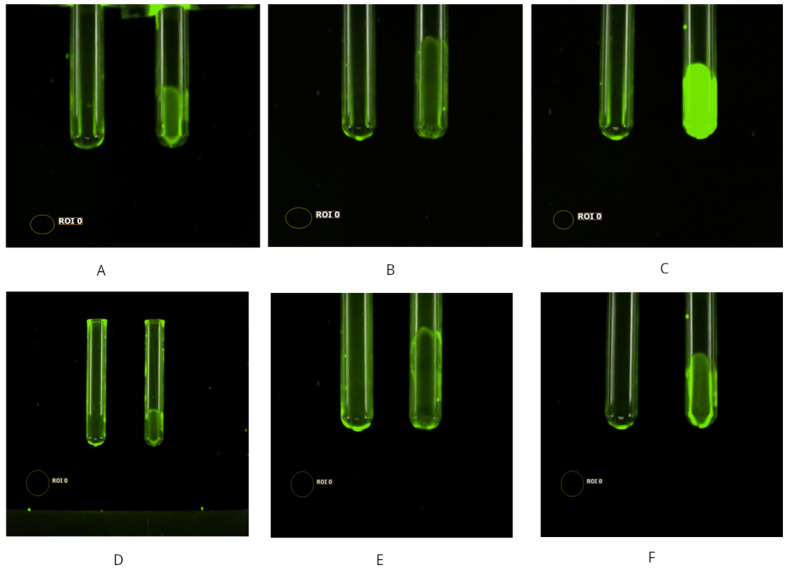
Blood images Ex 540 nm/Em 600 nm: empty nanoparticle carrier (**A**), epirubicin (**B**), and POLEPI (**C**). Blood images Ex 480 nm/Em 600 nm: empty nanoparticle carrier (**D**), epirubicin (**E**), and POLEPI (**F**).

A strong POLEPI signal was detected in the blood at 540 nm/600 nm (Figure 4A–C); however, an excitation/emission pair of 480 nm/550–600 nm demonstrated the best positive control (epirubicin) and negative control (empty NP) signal profiles across blood and tissue types. This excitation/emission channel pairing passed both the negative and positive control test conditions and was, thus, selected for Subpart B. The results on blood corroborate findings from Subpart A1, indicating that whole-body imaging reflects the blood distribution of epirubicin (Figure 4).

Localized and transient variations in vasculature dilation could potentially account for the seemingly variable distribution of signals at the whole-body level. Unlike Subpart A1, a poor signal was observed in the Ex 580 nm/Em 600 nm condition, likely due to the difference between in vivo and ex vivo imaging. A high background incongruent with quantification was noted in the Ex 540 nm/Em 550 nm condition. Workflow and image analysis led to an update to the workflow, where liquids (blood and bone marrow isolate) were routed to the plate reader for analysis.

### 2.3. Setting up of Optimal Conditions for Ex Vivo Imaging: Subpart B

**Figure 5:**
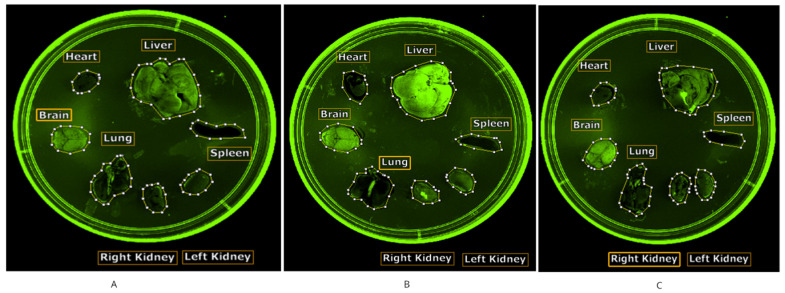
Tissue imaging Ex 480 nm/Em 550 nm: empty nanoparticle carrier (**A**), epirubicin (**B**), and POLEPI (**C**).

Subpart B aimed to assess the biodistribution of inherently fluorescent epirubicin, identifying an optimal exposure time of 6–6.7 s on the Newton 7 (Figure 5), and piloted final workflow for *n* = 45 in Subpart C. Liquids were imaged on the Varioskan for 1000 ms.

**Figure 6:**
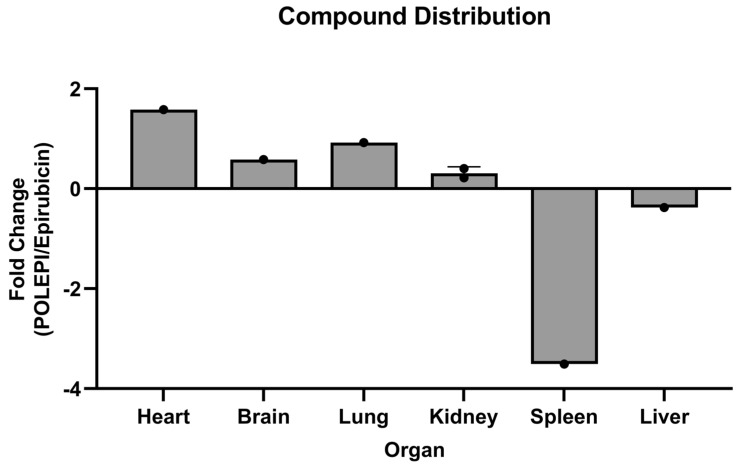
Fold change POLEPI/epirubicin by tissue.

Epirubicin levels were 3.5- and 0.38-fold higher in the spleen and liver, respectively (Figure 6). The distribution of POLEPI versus epirubicin revealed a distribution 1.58-fold higher in the heart, 0.58-fold higher in the brain, 0.92-fold higher in the lung, and 0.22-fold higher in the kidney, but 3.51-fold lower in the spleen and 0.38-fold lower in the liver (Figure 6).

**Figure 7:**
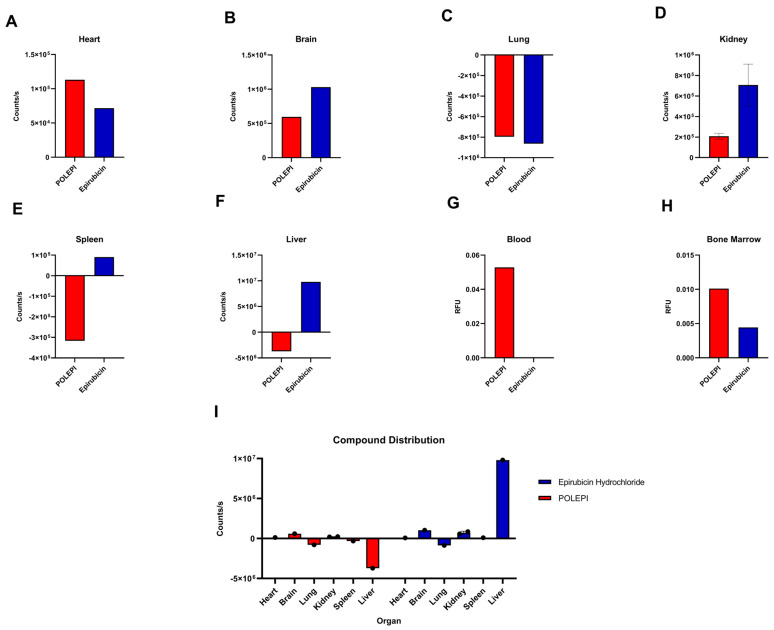
Tissues quantification counts/s or RFU: heart (**A**), brain (**B**), lung (**C**), kidney (**D**), spleen (**E**), liver (**F**), blood (**G**), bone marrow (**H**), and compound distribution (**I**).

In terms of counts per second, the liver was identified as the main target for epirubicin (but the lowest for POLEPI), followed by the brain, kidneys, and heart (Figure 7). For POLEPI, the brain exhibited the highest counts, followed by the kidney. However, counts per second for POLEPI were 2 logs lower in the heart than in the liver. The relative fluorescence units (RFU) were significantly higher in the blood of mice administered POLEPI.

### 2.4. Biodistribution of Epirubicin vs. POLEPI Measured Ex Vivo: Subpart C

**Figure 8:**
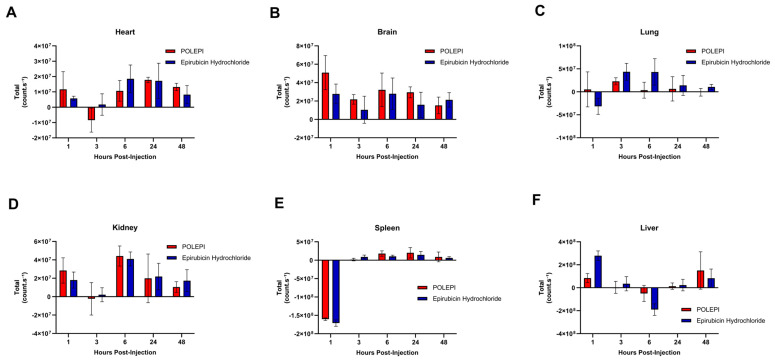
Biodistribution of POLEPI vs. epirubicin at different time points in tissues in non-tumored mice: heart (**A**), brain (**B**), lung (**C**), kidney (**D**), spleen (**E**), and liver (**F**). The data are expressed as mean ± SD.

The study was conducted in non-tumored animals to investigate the biodistribution of POLEPI compared to epirubicin and the empty NP vehicle control, via ex vivo optical imaging using Newton and Varioskan. The organs examined included the heart, brain, lung, kidney, spleen, liver, blood, and bone marrow at five different time points (1, 3, 6, 24, and 48 h) after a single intravenous dose (*n* = 3 mice/group). Both epirubicin and POLEPI were found to be distributed in the heart, brain, kidneys, spleen, and liver. In the heart (Figure 8A), the highest counts were observed for both POLEPI and epirubicin at 24 h, with no significant difference between them. In the brain, the highest POLEPI signal was detected 1 h post-injection (Figure 8B), indicating that POLEPI penetrated the brain more effectively than epirubicin. Conversely, POLEPI was not detected in the lungs (Figure 8C), whereas the highest epirubicin signal in the lungs was observed at 3–6 h post-dose. The highest signal for both POLEPI and epirubicin in the kidneys (Figure 8D) was observed at 6 h post-dose, with similar signal levels for both agents. Neither POLEPI nor epirubicin were detected in the spleen (Figure 8E). The liver was identified as the key target organ for both POLEPI and epirubicin (Figure 8F), with the epirubicin signal being highest immediately after dosing, while POLEPI persisted until 48 h. Both POLEPI and epirubicin were found in the tail snip immediately after dosing but were cleared by 6 h post-dose.

**Figure 9:**
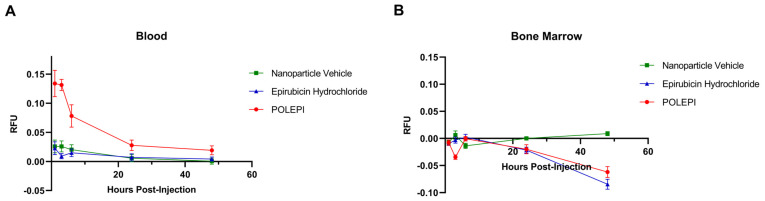
Biodistribution of POLEPI, epirubicin, and nanoparticle vehicle at different time points in tissues in non-tumored mice: blood (**A**) and bone marrow (**B**). The data are expressed as mean ± SD.

Overall, the fluorescence of POLEPI and epirubicin was found to be low in blood, with slightly higher fluorescence detected in the POLEPI-treated groups compared to the epirubicin-treated groups (Figure 9A). POLEPI detection peaked at 1 h post-dose and then declined over time at each time point. No fluorescence was detected in the bone marrow (Figure 9B). Interestingly, in many cases, the PBS control sample exhibited a higher background signal than the actual bone marrow samples. Neither POLEPI nor epirubicin was distributed in the bone marrow.

In summary, the liver emerged the key target organ for both agents, with POLEPI persisting longer in this organ compared to epirubicin. The kidneys were identified as the second target organ; however, no significant differences were observed between the two drugs in terms of distribution. A notable discrepancy in the distribution of POLEPI and epirubicin was observed in the lungs, with POLEPI failing to enter this organ. Although the amount of epirubicin delivered to the heart was similar for both drugs, POLEPI exhibited elevated levels for up 48 h. Additionally, POLEPI demonstrated superior and faster penetration of the brain compared to epirubicin. Neither POLEPI nor epirubicin infiltrated the spleen. The time points utilized in Subpart C were leveraged to study epirubicin biodistribution in tumor-bearing mice.

### 2.5. Epirubicin and POLEPI Distribution in Immunocompromised Mice Bearing Patient-Derived Ovarian Tumors

The objective of the study was to investigate whether the nanoparticle carrier affects the distribution of epirubicin to tumors and to compare the kinetics of POLEPI accumulation in tumors and normal tissues with epirubicin. The hypothesis was that POLEPI would remain in tumors longer, leading to more efficient cancer cell killing while sparing normal organs. Therefore, the biodistribution of POLEPI relative to epirubicin was assessed using the inherent molecular fluorescence of epirubicin in an ovarian orthotopic tumor model (CRT_OV_00367). Immunocompromised mice implanted with patient-derived ovarian tumors were utilized for the study. The distribution of epirubicin and POLEPI in various organs (heart, liver, brain, kidney, lung, spleen, tumor, and ovary) was evaluated ex vivo using the Newton imaging system at five different time points (1, 3, 6, 24, and 48 h) after a single dose.

**Figure 10:**
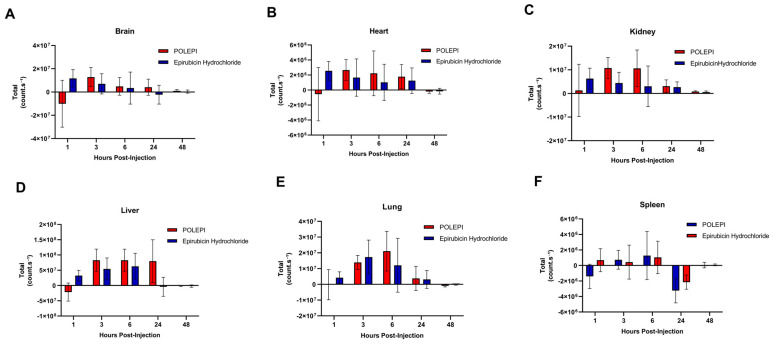
Biodistribution of POLEPI vs. epirubicin at different time points in tissues in tumored mice: brain (**A**), heart (**B**), kidney (**C**), liver (**D**), lung (**E**), and spleen (**F**). The data are expressed as mean ± SD.

The distribution of epirubicin in tumor-bearing mice was observed to occur relatively quickly, with penetration into organs detected as early as 1 h post-dose. In contrast, POLEPI took longer to disseminate into the organs but delivered higher amounts of epirubicin to various organs, including the brain, heart, kidney, and liver, over the course of 48 h (Figure 10).

**Figure 11:**
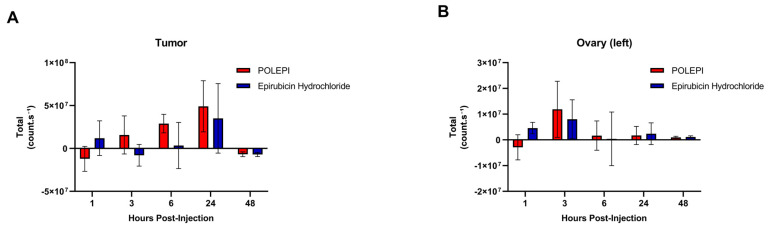
Biodistribution of POLEPI vs. epirubicin in the tumor (**A**) and left ovary (**B**) at different timepoints in tumored mice. The data are expressed as mean ± SD.

**Figure 12:**
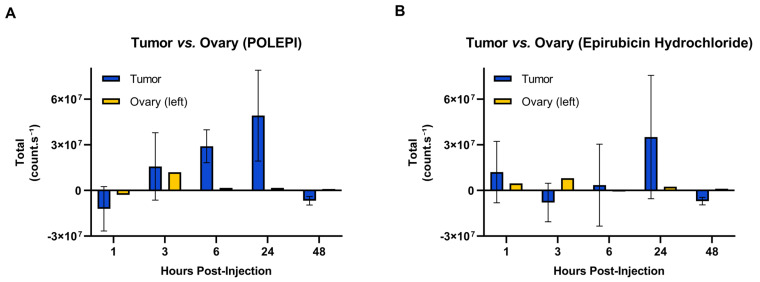
POLEPI (**A**) and epirubicin (**B**) distribution in the tumor compared to the ovary. The data are expressed as mean ± SD.

Distinct differences were noted in the biodistribution of POLEPI within the tumor compared to epirubicin (Figure 11A). POLEPI exhibited accumulation starting at 3 h post-dose, reaching a peak at 24 h. Over the 48 h period, a significantly higher accumulation of counts/s was observed in the tumor following POLEPI administration compared to epirubicin. However, both POLEPI and epirubicin appeared to be cleared from the tumors after 48 h. Furthermore, both POLEPI and epirubicin were cleared from the healthy left ovary by 6 h post-dose (Figure 11B). Interestingly, compared to the left healthy ovary, there were higher counts of both POLEPI and epirubicin observed in the tumor (Figure 12), indicating preferential uptake of POLEPI within the tumor tissue.

Although the direct comparison of biodistribution between non-tumor-bearing and tumor-bearing animals may not be entirely reliable due to the differing exposure times, certain distinctions are evident. In tumor-bearing mice, unlike epirubicin, POLEPI was distributed in all tissues by 3 h post-dose, with practically no detectable fluorescence signal at 48 h for either epirubicin or POLEPI. This contrasts with non-tumor animals, where the epirubicin signal from both epirubicin and POLEPI was still measurable at 48 h.

Notably, in the hearts of tumor-bearing animals, the total fluorescence counts were approximately one order of magnitude lower than those observed in non-tumor-bearing mice (Figure 8A and Figure 10B). POLEPI began to appear at the 3 h time point and gradually declined to zero by 48 h, whereas in non-tumored animals, it remained elevated at 48 h.

In both non-tumored and tumored animals, the liver serves as the primary target organ. However, in tumored animals, the fluorescence counts observed in the liver are approximately one order of magnitude lower than those observed in non-tumor mice. Notably, unlike those of epirubicin, the liver levels of POLEPI remain consistently higher from 3 to 24 h post-injection (Figure 8F and Figure 10D).

In the brains of tumor-bearing animals, florescence counts were higher in mice administered with POLEPI compared to epirubicin-injected mice. However, these counts declined from 3 to 48 h post-injection, whereas they remained at higher levels from 1 to 48 h in non-tumored animals (Figure 8B and Figure 10A).

Additionally, POLEPI persisted longer in the lungs of tumor-bearing animals compared to non-tumor-bearing animals (Figure 8C and Figure 10E).

In the spleen, there was no significant difference in the biodistribution of epirubicin and POLEPI observed between non-tumored and tumored animals. Neither compound was detected in the spleen, indicating a consistent lack of distribution to this organ across both groups (Figure 8E and Figure 10F).

In the kidney, the biodistribution of POLEPI exhibited differences between tumored and non-tumored animals. In non-tumored mice, POLEPI counts increased from 1 to 6 h post-injection and then declined over time. Comparatively, in tumored animals, counts increased from 3 to 6 h post-injection and then declined thereafter (Figure 8D and Figure 10C).

**Figure 13:**
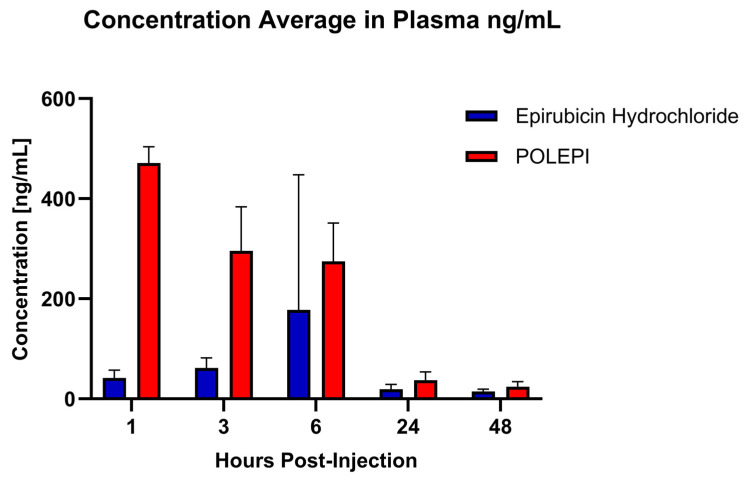
Biodistribution of POLEPI vs. epirubicin in the plasma at different timepoints in tumored mice. The data are expressed as mean ± SD.

In plasma samples obtained from the orthotopic ovarian tumor model (CRT_OV_00367), the LC-MS/MS analysis revealed distinct differences in the biodistribution of POLEPI compared to epirubicin at various time points post-dose. The highest concentration of epirubicin was detected at 6 h post-dose, with smaller amounts observed at other time points. Conversely, the highest concentration of POLEPI was detected at 1 h post-dose, indicating a faster distribution into the bloodstream compared to epirubicin. Importantly, the amount of POLEPI detected in plasma samples was consistently higher than that of epirubicin across all time points assessed (Figure 13).

## 3. Discussion

EPI delivery in nanosystems has been an interesting strategy to overcome cardiotoxicity and myelosuppression and improve the safety and efficacy of EPI. A crucial aspect of the translational evaluation of nanomedicines involves assessing the biodistribution of nanoparticles after in vivo administration in animals and humans. Various techniques have been developed for this purpose, such as histology, electron microscopy, liquid scintillation counting (LSC), the indirect measurement of drug concentrations, in vivo optical imaging, computed tomography (CT), magnetic resonance imaging (MRI), nuclear medicine imaging, and light sheet fluorescence microscopy (LSFM) [6,10,11]. In particular, in vivo optical imaging is favored for its direct, non-invasive nature and relatively simple execution. In their analysis, Kumar et al. examined datasets from 2018 containing published pharmacokinetics of nanoparticles in plasma, tumors, and 13 different tissues of mice. They focused on the biodistribution characteristics of various nanoparticles, including graphene oxide, lipid, polymeric, silica, iron oxide, and gold nanoparticles, and quantitatively characterized them using nanoparticle biodistribution coefficients (NBC) [12]. The data points were categorized into early (0–6 h), middle (6–72 h), and late (greater than 72 h) time points. Their observations indicated that, at early time points, nanoparticles primarily distributed in organs such as the spleen, liver, intestine, lungs, heart, kidneys, and tumor tissues. The highest concentrations of nanoparticles were observed in the blood (10.4% ID/g), spleen (10.8% ID/g), and liver (19.7% ID/g). As time progressed, nanoparticles were rapidly cleared from the blood (NBC decreased from 10.3% ID/g at early time points to 0.4% ID/g at later time points) and gradually distributed into the intestine (NBC increased from 1.7 to 2.6% ID/g). At later time points, there was a tendency for nanoparticles to accumulate predominantly in the liver (13.2% ID/g) and spleen (15.9% ID/g). Broginni et al. investigated the serum and tissue distribution of epirubicin following intravenous injection of 15 mg/kg in C57BL mice with a 14-day intramuscular Lewis lung carcinoma [13]. They measured total fluorescence and quantified unchanged drugs by separating them using thin-layer chromatography combined with scanning fluorescence. In the serum, the disappearance of epirubicin accounted for less than 50% of the total fluorescence as early as 30 min after drug injection. In tissues, the measured fluorescence was predominantly attributed to the native compounds, suggesting uptake of the drug by the tissues.

Notably, levels of epirubicin were significantly lower than those of DX in the tumor and spleen, while the concentrations of the two isomers were comparable in the heart, liver, and kidneys. Numerous doxorubicin nanocarrier formulations, such as aldehyde dextran-based nanoparticles and hypoxia-responsive carboxymethyl dextran, have demonstrated enhanced tumor penetration compared to doxorubicin [14,15,16]. Among epirubicin nanoparticles, NC-6300 has progressed to clinical trials, representing a molecule where epirubicin is covalently bound to a polyethylene-glycol polyaspartate block copolymer through an acid-labile hydrazone bond [17]. Pharmacokinetic studies in rats showed significantly improved plasma retention of NC-6300 compared to native epirubicin.

Tissue distribution studies revealed efficient epirubicin release in tumors, reaching 74% by area under the concentration–time curve (AUC) evaluation. NC-6300 exhibited inhibitory effects on the growth of MDA-MB-231 human breast tumors and regressed Hep3B human hepatic tumors in vivo. Moreover, it demonstrated reduced cardiotoxicity compared to conventional epirubicin formulations, as assessed by echocardiography [18].

In a phase 1 study with patients having advanced or recurrent solid tumors (*n* = 19), NC-6300 demonstrated safety, tolerability, and efficacy. The maximum tolerated dose (MTD) and recommended phase 2 dose (RP2D) were determined to be 170 mg/m^2^. Stable disease was observed in 10 patients, and a partial response was noted in one patient with breast cancer [19]. Subsequently, a phase 1b trial in patients with advanced, metastatic, or unresectable solid tumors (*n* = 29) confirmed the well-tolerated nature of NC-6300, establishing MTD at 185 mg/m^2^ and RP2D at 150 mg/m^2^ [20]. The study revealed an 11% objective response rate, including partial responses in angiosarcoma and endometrial stromal sarcoma cases.

An expansion cohort study focusing on angiosarcoma (*n* = 10) showed promising activity of NC-6300, with a median progression-free survival (mPFS) of 5.4 months. In subjects without prior anthracycline treatment, mPFS reached 8.2 months, indicating potential efficacy in this subpopulation. NC-6300 maintained a well-tolerated profile throughout these trials [21].

Matin et al. investigated the biodistribution of epirubicin loaded onto superparamagnetic iron oxide nanoparticles (SPION) coated with mesoporous silica nanoparticles (MSN). This novel formulation, which also included an anti-miR-21-expressing plasmid and a zeolitic imidazolate framework (ZIF-8), specifically targeted colon adenocarcinoma. The combination demonstrated enhanced therapeutic efficacy, with improved delivery and retention in tumor tissues while minimizing systemic toxicity [22].

In our research, we observed that POLEPI exhibited significantly superior distribution to orthotopically grafted ovarian patient-derived xenografts (PDX) compared to epirubicin. The concentration, as indicated by the fluorescence signal, was 29-fold higher in the targeted PDX than in the adjacent healthy ovary and two-fold higher than that of epirubicin at 24 h post-injection. Our recently published preliminary findings regarding antitumor activity revealed that POLEPI outperformed EPI in terms of tumor volume reduction. However, unexpectedly, off-target toxicity was observed using the same orthotopic PDX model [23]. This swift and substantial accumulation of polysaccharide-based nanoparticles, even without specific homing molecules, suggests a potential “per se” targeting mechanism involving the saccharide shell and cellular membrane glucose transporters (GLUT).

Dextran-based nanocarriers, such as POLEPI, have been reported to exhibit an affinity for GLUT [24]. Cancer cells, characterized by an overexpression of GLUT, experience an increased demand for glucose. The Warburg effect, proposed by Otto Warburg in 1924, theorizes that cancer cells shift their glucose metabolism to a less efficient glycolysis process, leading to heightened glucose demand. This metabolic alteration is exploited as a potential targeting mechanism for cancer treatment due to the significantly increased glucose consumption by cancer cells compared to normal cells.

In our study, the rapid and robust accumulation of POLEPI could be partially attributed to metabolic targeting, leveraging the increased glucose demand characteristic of cancer cells. This phenomenon enhances the specificity of nanoparticle delivery to cancerous tissues, contributing to the potent accumulation observed.

## 4. Materials and Methods

All studies in nude mice were performed by Certis Oncology Solutions (4940 Carroll Canyon Road, Suite 120, San Diego, CA, USA).

### 4.1. Animals

Nude female 6–12 weeks old mice were obtained from Taconic Biosciences (Germantown, NY, USA) or Jackson Laboratories (Bar Harbor, ME, USA). Animal welfare followed the U.S. Department of Agriculture’s Animal Welfare Act (9 CFR Parts 1, 2, and 3) as applicable. The study protocol was approved by the Institutional Animal Care and Use Committee (IACUC) ACUP EB17-030. The study protocol and supporting documentation involving animal procedures were reviewed and approved by the Institutional Animal Care and Use Committee (IACUC) of the Explora Biolabs. The study was conducted in accordance with the regulations of the Association for Assessment and Accreditation of Laboratory Animal Care (AAALAC) of Explora Biolabs. Certis Oncology is pursuant to the Clinical Laboratory Improvement Amendments of 1988 (“CLIA”) and accompanying regulations.

### 4.2. Test Article Preparation and Animal Dosing

Epirubicin hydrochloride (Ellence, NDC 0009-5091-01, Pharmacia & Upjohn Company LLC, Division of Pfizer Inc., New York, NY, USA) was purchased from Pfizer as a 2 mg/mL solution for injection into saline. POLEPI batch NV-Epi-222 was supplied as a lyophilized powder containing 3.32% epirubicin HCl (Supporting Information Table S1), and nanoparticle carrier batch NV-carrier-221 containing 1.2 g per vial. For each study subpart, a fresh dosing solution was prepared by dissolving POLEPI powder in water for injection (WFI) to deliver 2 mg/mL (eq) solution (Supporting Information Table S2). The animals were intravenously administered 12.5 mg/kg (epirubicin eq) (6.25 mL/kg). For Subpart C, the epirubicin HCl content in batch NV-Epi-222 was 3.32%, but calculations were made based on 4.3%, erroneously. This difference was considered negligible for these studies. A dose solution of 1.53 mg/mL (EPI eq), as opposed to 2 mg/mL (EPI eq), was prepared. This translates to dosing at 9.65 mg/kg (eq) as opposed to 12.5 mg/kg (eq). NP vehicle (negative control) was rehydrated with WFI to provide 60.24 mg/mL NPs solution (Supporting Information Table S3). A total of 376.5 mg NP/kg was administered to each mouse.

### 4.3. Orthotopic Engrafting of Certis Oncology Ovarian PDX for POLEPI and Epirubicin Biodistribution Evaluation

CRT_OV_00367 represents a serous ovarian cancer sample derived from bowel metastasis in a female patient who had previously undergone treatment with carboplatin, paclitaxel, doxorubicin, olaparib, topotecan, gemcitabine, and bevacizumab. For experimental purposes, female NOG mice (Taconic, Germantown, NY, USA) were subcutaneously implanted with CRT_OV_00367 tumors and monitored until the tumors reached a volume of 500–1000 mm^3^. Subsequently, the tumor tissues were collected and dissociated into 3 × 3 tumor chunks. To establish recipient mice (*n* = 112) for further investigation, animals were anesthetized with isoflurane, shaved, and surgically prepped using surgical scrub and 70% isopropyl alcohol. An incision was made below the right rib cage, and the peritoneal wall was gently bluntly dissected. Following an incision in the peritoneal wall over the ovary, the ovary was carefully removed from the cavity. Tumor fragments were then sutured to the ovaries, and the peritoneum was sealed with sutures. Closure of the skin was achieved using wound clips, and analgesics were administered for postoperative care. Interrupted sutures were placed at the incision site. Postoperatively, the animals were regularly imaged using Aspect Imaging M3 MRI (magnetic resonance imaging) to monitor tumor growth. Tumor volume was quantified using VivoQuant 2021 software (Invicro, Needham, MA, USA). Upon reaching tumors measuring 174–386 mm^3^ with an average of 256 mm^3^ in 75 animals, they were randomly assigned to their respective treatment groups and dosed within 48 h. Animals were injected with epirubicin hydrochloride (12.5 mg/kg) and POLEPI (12.5 mg/kg eq.), or empty NP vehicle (376.5 mg/kg) once intravenously for five time points (1, 3, 6, 24, and 48 h) imaging. There were 15 groups (*n* = 5 mice/group): empty NP carriers, epirubicin hydrochloride (Ellence), and POLEPI. The organs were collected at specific time points after a single dose. Ex vivo organs were imaged via Newton 7.0 (Vilber, Collégien, France) at Ex 480 nm/Em 550 nm (settings determined in Subpart B), immediately snap-frozen, and stored at −80 °C at Certis. The images were quantified using fluorescence. However, a shorter (1–2 s) exposure time than that in the Subpart C study (5–6 s) was used. Thus, it is difficult to compare a tumor-bearing study vs. a non-tumor study by directly comparing absolute counts/organs.

### 4.4. Determination of Imaging Conditions Using Newton 7.0 Imager

The study was divided into four subparts to first set the optimal imaging conditions.

The goal of Subpart A1 was to identify the peak inherent fluorescence of epirubicin using whole-body in vivo imaging of nude mice over a 48 h period to determine the imaging conditions for Subpart A2. Naïve mice were injected with saline (*n* = 1) or epirubicin (*n* = 1). Whole animals were imaged side by side using Newton 7.0 imager at 10 min, 30 min, 1 h, 3 h, 6 h, 24 h, and 48 h post-dose to identify the peak fluorescence time point.

The purpose of Subpart A2 was to identify the optimal Ex and Em channels and exposure times for epirubicin and POLEPI in ex vivo organs and blood. One naïve animal per group was injected with NP vehicle, epirubicin, or POLEPI. Between 1 and 4 h post-injection, blood was collected from the mice, and they were anesthetized for perfusion. Following perfusion, the mice were sacrificed, and the liver, heart, spleen, and kidney were harvested. Blood from each mouse was collected, and 100 µL was transferred into an Eppendorf tube and imaged with the cap open/removed, side by side, with an empty Eppendorf tube with the cap open/removed to define the background signal. The liver, heart, spleen, and kidney from each mouse were placed together in an open petri dish and imaged side by side with an empty open petri dish to define the background signal. The organs from each mouse were imaged at the excitation and emission wavelengths (nm). The exposure time and post-dose time point were recorded: Ex 480/Em 550, Ex 480/Em 600, Ex 540/Em 550, Ex 540/Em 600, Ex 580/Em 550, and Ex 580/Em 600. If strong signals were not observed in these channels, then other Ex/Em combinations were used.

Subpart B was designed to identify the full-time workflow from harvesting to imaging completion to set the schedule for Subpart C. One naïve animal per group was injected with NP vehicle, epirubicin, or POLEPI. At 1 h post-injection, blood was collected from each animal, and then, the mice were anesthetized, perfused with saline, and sacrificed to facilitate the collection of organs. The rinsing with saline was performed gently to avoid non-physiological “pushing” of epirubicin or POLEPI into the vascular placenta, such as the spleen or liver. The time from harvest start to imaging/file saving end was recorded to facilitate dosing and harvesting cadence for subproject C. Blood was collected from each animal and transferred to an EDTA tube. The tube was inverted to fully mix blood and EDTA. Fifty microliters were plated in triplicate per animal, resulting in three wells per animal and nine wells in total. The plates were read on a Varioskan^®^ Lux microplate reader (Thermo Fisher, Waltham, MA, USA, Model #N7-00020) with excitation and emission of Ex 480 nm/Em 550 nm (1000 ms). Bone marrow was collected in a conical tube during saline flushing. The conical tube was spun down, and the bone marrow was resuspended in PBS. Fifty microliters were plated in triplicate per animal, resulting in three wells per animal and nine wells in total. The plate was read using a Varioskan with excitation and emission of Ex 480 nm/Em 550 nm (1000 ms). Organs from a single animal were placed together in an open Petri dish and imaged side by side with an empty, open Petri dish to define the background signal. One or more Petri dishes were used to facilitate proper distancing between the organs. The following tissues were collected: blood, brain, heart, liver, kidneys, lungs, spleen, and bone marrow (tibias and femurs were flushed with saline).

Subpart C was designed to establish the ex vivo time course of epirubicin/POLEPI fluorescence in nontumor-bearing nude mice. Naïve animals were intravenously injected with the NP vehicle (*n* = 3), epirubicin (*n* = 3), or POLEPI (*n* = 3). Blood samples were collected from each animal and transferred to an EDTA tubes. Fifty microliters were plated in triplicate per animal, resulting in three wells per animal and nine wells in total. The plate was read using a Varioskan with excitation and emission wavelengths set at Ex 480 nm/Em 550 nm (1000 ms). Bone marrow was collected in a conical tube during saline flushing. The conical plate was spun down, and the bone marrow was resuspended in PBS. Fifty microliters were plated in triplicate per animal, resulting in three wells per animal and nine wells in total. The plate was read using a Varioskan with excitation and emission set at Ex 480 nm/Em 550 nm (1000 ms). The following organs were collected, placed in an appropriate container, and imaged at 1, 3, 6, 24, and 48 h post-dose via Newton, before images were quantified to determine the level of fluorescence: blood, brain, heart, liver, kidney, lung, spleen, and bone marrow (tibias and femurs flushed with saline). After imaging, ex vivo organs were immediately snap-frozen and stored at −80 °C.

### 4.5. Quantification of Epirubicin in Plasma by HPLC/MS/MS

The LC-MS/MS bioanalytical method for quantifying epirubicin in mouse plasma underwent evaluation by processing and analyzing one set of calibration standards at both the front and back ends of plasma samples. Method accuracy was assessed by comparing measured accuracies with back-calculated concentration values for the calibration standards. Method precision was determined through the relative standard deviations of these values. Acceptance criteria were defined as an average accuracy within ±30% for the lowest calibrator and ±25% for all other calibrators.

Preparation of Plasma Calibration Standards: Eleven calibration standards of epirubicin were prepared in plasma for quantitation. Plasma calibration standards were prepared as follows: 47.5 µL vehicle control plasma was transferred to wells A1–A12 of a 1 mL round-bottom 96-well microtiter plate. Next, 2.5 µL of each of the above sub-stocks were spiked into successive wells of the same microtiter plate. Specifically, sub-stock solution 12 was spiked into well A1, sub-stock solution 11 was spiked into well A2, and so on, until sub-stock solution 1 was spiked into well A12.

Processing of plasma samples: A total of 75 plasma samples were analyzed, with five samples at each of the five time points (1, 3, 6, 24, and 48 h) for three groups. Five samples were used for calibration curves. One sample from each vehicle time-point was pooled to create a matrix calibration curve.

These samples were retrieved from the −20 °C freezer, thawed at room temperature, and then, a 50 µL aliquot of each sample was transferred to the available wells of the same 1 mL round-bottom microtiter plate used for the calibration standards. These samples were placed in wells B1–H3 of a plate. To each well of the microtiter plate containing either the plasma calibrator or plasma study sample, 200 µL of ice-cold acetonitrile containing a proprietary internal standard (SciAnalytical Strategies) at a concentration of 200 ng/mL was added. The internal standard was selected based on similar chromatographic retention times and similar molecular weights to the epirubicin test article. The samples were then placed on the deck of a Hamilton Nimbus 96-channel liquid handler. Samples were extracted by repetitive pipetting using a wide-bore pipette tip, to ensure excellent mixing of the suspended plasma/acetonitrile solution. After 5 min of repetitive pipetting/mixing, the samples were drawn into the same wide-bore tip, which was then robotically inserted into a tightly fitting filter tip. The filter allowed the filtrate to pass through cleanly and was collected in a shallow-well, v-bottom 96-well microtiter plate. A 100 µL aliquot of the collected filtrate was then transferred to an analysis plate, diluted 1:1 with water containing 0.1% formic acid, and placed on the autosampler for LC/MS/MS analysis.

### 4.6. Data Processing and Statistical Analysis

Certis Oncology managed all the data using the StudyLog v3.1.399 data management software.

Background Subtraction: Control group (empty NP) signals were subtracted from treated group signals in cases with *n* = 1. For cases with *n* = 2 (e.g., kidneys), the average measurement of the control group was subtracted from the treatment measurement individually (i.e., epirubicin kidney signal minus average control kidney signal).

Fold Change Calculation: The fold change was calculated using background subtracted values by dividing the treatment by the reference, according to the specifics of the subpart:

Subpart A1: Saline/Epirubicin = Fold Change

Subpart A2: Empty Nanoparticle/Epirubicin = Fold Change

Subpart B: Empty Nanoparticle/POLEPI = Fold Change

Subpart C: Epirubicin/POLEPI = Fold Change

The imaging parameters determined for each subpart are shown in Table 1. The statistical analysis and graphs were performed using GraphPad Prism 9.0.0 software [25]. Data are expressed as the mean ± SD (standard deviation) and are not statistically significant.

## 5. Conclusions

Our studies reveal that polysaccharide-based nanoparticles (POLEPI) show significantly enhanced accumulation and retention in tumor tissue compared to epirubicin hydrochloride (EPI). While whole-body imaging of epirubicin’s inherent fluorescence using the Newton 7.0 imager proved impractical, ex vivo imaging demonstrated that the distribution of POLEPI to orthotopically engrafted ovarian patient-derived xenografts (PDX) was notably faster than that of epirubicin.

At 24 h post-injection, POLEPI achieved a striking 29-fold higher fluorescence signal in the tumor compared to the adjacent healthy ovary, whereas the EPI group showed a 15-fold increase. This result indicates a clear preferential uptake of POLEPI in the tumor. The difference became even more pronounced at 6 h post-injection, with a remarkable 17-fold increase in the POLEPI group versus a 9-fold increase in the EPI group. Additionally, at this time point, the fluorescence signal of POLEPI was twice as high as that of epirubicin, underscoring POLEPI’s superior targeting and retention capabilities.

We further demonstrated the feasibility and utility of a cost-effective, rapid method for biodistribution analysis by leveraging the inherent fluorescence of epirubicin. Although fluorescence imaging has inherent limitations compared to HPLC/MS methods in its ability to distinguish the parent drug from its metabolites, it remains a practical and efficient approach for initial biodistribution assessments. This method is particularly advantageous for proof-of-concept preclinical studies, where high throughput and cost efficiency are critical considerations. To obtain a complete pharmacokinetics profile, future studies incorporating validated analytical techniques such as HPLC/MS are recommended. These methods will provide a more precise and comprehensive analysis of drug distribution, metabolism, and pharmacokinetics, thereby further validating and expanding upon these findings to meet regulatory requirements.

## Figures and Tables

**Table 1:** Imaging parameters for each Subpart and PDX model.

Subpart	Channels	Exposure Times
A1	Ex580/Em600, Ex540/Em600	1–5 s
A2	Ex480/Em550, Ex480/Em600 Ex540/Em550, Ex540/Em600 Ex580/Em550, Ex580/Em600	5 s
B	Tissue: Ex480/Em550 Blood: Ex480/Em550	6 s 1000 ms
C	Tissue: Ex480/Em550 Blood: Ex480/Em550	5–6 s 1000 ms
Orthotopic Ovarian PDX	Tissue: Ex480/Em550	1–2 s

PDX, patient-derived xenografts; Ex, excitation wavelength; Em, emission wavelength.

## Data Availability

The data presented in this study are available upon request.

## Supplementary Materials

The following supporting information can be downloaded at: https://www.mdpi.com/article/10.3390/ijms26030970/s1. References [7,26,27,28,29] are cited in the Supplementary Materials.
