# Supplementary material for: Biodistribution of Polyaldehydedextran Nanoparticle-Encapsulated Epirubicin in Ovarian Tumor-Bearing Mice via Optical Imaging
Source: Int J Mol Sci. 2025 Jan 24;26(3):970. doi: 10.3390/ijms26030970 (PMC11817601; doi:10.3390/ijms26030970)
Supplement: Supplementary file 1 [file ijms-26-00970-s001.zip › ijms-3352479-supplementary.pdf]

Supporting Information

Biodistribution of Polyaldehydedextran Nanoparticle-Encapsulated Epirubicin in Ovarian Tumor-Bearing Mice via Optical Imaging

Wioletta Kośnik [1,], Hanna Sikorska [1], Adam Kiciak [2] and Tomasz Ciach [1,2,3,]**

[1] NanoVelos S.A., Rakowiecka 36, 02-532 Warsaw, Poland

[2] NanoGroup S.A., Rakowiecka 36, 02-532 Warsaw, Poland

[3] Faculty of Chemical and Process Engineering, Warsaw University of Technology, Waryńskiego 1, 00-645 Warsaw, Poland

*Correspondence: w.kosnik@nanogroup.eu (W.K.); tomasz.ciach@pw.edu.pl (T.C.)

1. Synthesis

1.1. Synthesis of polyaldehydodextran

The dextran (70 kDa, pharmacopeia grade, Pharmacosmos, Holbaek, Denmark) was oxidized according to a modified protocol of Muangsiri and Fuentes [26,27]. Briefly, 25.5 g of dextran was dissolved in 500 mL ultra-pure water (Type 1; conductivity < 1 µS/cm at 25°C, resistivity 18 MΩ-cm at 25°C). Subsequently, 6.73 g sodium metaperiodate (Sigma-Aldrich) was added in molar ratios of 1:10, (IO_4^-/glucose units) to obtain approximately 14% (13,78%) of glucose ring oxidation. The solution was stirred in the dark at room temperature for 1 h. The post-reaction solution was purified against distilled water using a tangential flow filtration (GAMBRO, Polyflux 14L). After two hours of filtration, the titration method (iodide & iodate method) was used to confirm the absence of iodates in the wastewater. The final product was freeze-dried or spray-dried which allowed obtaining the product in the form of a white solid. The number of aldehyde groups in polyaldehydodextran (PAD) was determined using a modified hydroxylamine hydrochloride method [28] with unmodified dextran as a reference. The final product was stored in the refrigerator in a sealed container.

1.2. Synthesis of dextran nanoparticles with epirubicin (Epi-NPs)

Dextran nanoparticles with epirubicin were prepared according to the preparation method of nanoparticles from polysaccharides as described in the earlier publication [7] using dextran with a molecular weight of 70 kDa (oxidation degree ~14%) and dodecylamine hydrochloride as a coiling agent. An example of the procedure for the synthesis of dextran nanoparticles with epirubicin: 1,5 g of dried PAD (polyaldehyde dextran) was dissolved in 15 ml of ultra-pure water (Type 1; conductivity < 0,1 µS/cm at 25°C, resistivity 18 MΩ-cm at 25 ^{o}C) at 30°C, and 15,0 mL of 1% solution of epirubicin hydrochloride was added. Then 4,044 mL of 2% solution of dodecylamine hydrochloride was added. Before using all solutions of reagents were warmed to 30 °C. The mixture was constantly stirred at 30 °C for 30 min (magnetic stirrer, 350 rpm). The pH was measured and increased with a 0.1 M sodium hydroxide solution within 60 min until the solution reached pH 9. At the time 4,314 mL of 4% solution of alanine was added.

Finally, the pH of the mixture was decreased to 7.4 with 0.1 M HCl solution. The final product was lyophilized which allowed obtaining the product in the form of an orange-red solid.
Epi-NPs was prepared with an average size of 100-200 nm as measured in aqueous solutions using NanoSight LM 10 (405 nm laser). The determined epirubicin content in a dry matter of nanoparticles is approximately 4%. The obtained nanoparticles were freeze-dried and stored in sealed containers at a temperature of 2-8 °C.

1.3. Synthesis of empty dextran nanoparticles

An example of the procedure for the synthesis of dextran nanoparticles: 6,4 g of dried PAD was dissolved in 64 ml of ultra-pure water at 16.7 mL of 2% solution of dodecylamine hydrochloride was added. Before using all solutions of reagents were warmed to 30°C. The mixture was constantly stirred at 30°C for 30 min. The pH was measured and increased with a 0.5 M sodium hydroxide solution within 60 min until the solution reached pH 9. At the time 19.0 mL of 4% solution of alanine was added. Finally, the pH of the mixture was decreased to 7.4 with 0.5M HCl solution. The final product was lyophilized.
NPs were prepared with an average size of **137.2 ± 4.3 nm** as measured in aqueous solutions using NTA and DLS. The obtained nanoparticles were freeze-dried and stored in sealed containers at a temperature of 4 °C

1.4. Determination of epirubicin hydrochloride concentration in NV-Epi-222 (POLEPI) based on calibration curve and by means of UV-Vis spectroscopy

Three random stock solutions of NV-Epi-222 were prepared in pre-warm and sterile PBS. Samples were preapared to contain at maximum 7,0mg of nanocarrier. Each of three samples was dissolved in 1ml of sterille and pre-warm PBS. In order to dissolve the compound completely, the solutions were mixed for ca. 30min. From each prepared stock solution a portion of 100μl was collected and dissolved in 2,9ml of PBS in order to obtain a dilution apropiate for UV-Vis measurements. Next, for each final sample UV-Vis absorption spectra was measured in the range 200-1000nm in quartz cuvettes with cell path length of 1cm. Absorption band at λmax=480nm was used as the reference one for calculation of the EPI HCl concentration.

Table S1. The mass and percantage contribution of epirubicin hydrochloride in NV EPI 222 samples.

Sample number	NV Epi 222 weight [mg]	Abs at λ_{max}= 480nm (solution: 100µl stock sample + 2,9 ml of PBS)	Epi HCl concentration in measured sample [mM]	Epi HCl concentration in stock sample [mM]	Epi HCl weight [mg]	**% Epi HCl in NV Epi 222**
1	3,2	0,077	$6,39*10^{-3}$	0,1917	0,111	**3,47%**
2	6,5	0,134	$1,20*10^{-2}$	0,2089	0,209	**3,21%**
3	4,8	0,104	$9,05*10^{-3}$	0,2715	0,157	**3,28%**
						**Average 3,32%**

1.5. Procedure of preparation of Stock Solution of Epi HCl (epirubicin hydrochloride) in NV-Epi-222 (POLEPI)

NV-Epi-222: each vial contains 1,01g of POLEPI with 3,32% of Epi HCl what gives 33,53mg of the active compound.

PBS (calcium and magnesium-free for cell culture) for NV-Epi-222 dissolution

Table S2. Preparation of POLEPI stock solution

Parameter	Value	Unit
Epi HCl Molecular weight [g/mol]	580	g/mol
NV-Epi-222 sample weight [g]	1,01	g
% Epi HCl	3,32%	
Epi HCL weight in NV-Epi-222 [g]	0,033532	g
Epi HCL weight in NV-Epi-222 [mg]	33,532	mg
Stock concentration Epi HCl in NV-Epi-222 [mg/ml]	2	mg/ml
Stock concentration of NV-Epi-222 [mg/ml]	60,24	mg/ml
volume of PBS for NV-Epi-222 dissolution [ml]	16,766	ml
volume of PBS for NV-Epi-222 dissolution [µl]	16766	µl
volume of PBS for NV-Epi-222 dissolution [l]	0,016766	l
mol number of Epi HCl in NV-Epi-222 [mol]	5,78138E-05	mol
stock solution of Epi HCl (in NV-Epi-222) [mol/l]	0,00345	mol/l = M
stock solution of Epi HCl (in NV-Epi-222) [mmol/l]	3,45	mmol/l = mM

1,01 g (1 vial) of NV-Epi-222 was dissolved in 16 ml and 766 µl of sterile and pre-warm PBS, to obtain 2 mg/ml (3,45 mM) stock solution of Epi HCl captured in NV-Epi-221 (60.24mg/ml POLEPI solution). The stock solution was then used to prepare a series of dilutions. For dilutions, a cell medium was used. The type of cell medium was choose based on the type of cell culture that was involved in ex-vivo studies.

1.6. Procedure of preparation of Stock Solution of NV-carrier-221(empty NPs)

NV-carrier-221: each vial contains 1,23g of blank nanoparticle

PBS (calcium and magnesium-free for cell culture) for NV-carrier-221 dissolution

Table S3. Preparation of empty NPs stock solution

Parameter	Value	Unit
NV-carrier-221 sample weight [g]	1,23	g
NV-carrier-221 sample weight [mg]	1230	mg
Stock concentration of NV-carrier-221 [mg/ml]	60,24	mg/ml
volume of PBS for NV-carrier-221 dissolution [ml]	20,418	ml
volume of PBS for NV-carrier-221 dissolution [µl]	20418	µl

1,23 g (1 vial) of NV-carrier-221 was dissolved in 20 ml and 418 µl of sterile and pre-warm PBS to obtain 60,24 mg/ml stock solution. The stock solution was then used to prepare a series of dilutions. For dilutions, a cell medium was used. The type of cell medium was choose based on the type of cell culture that was involved in ex-vivo studies.

2. *In vitro* drug release

Epirubicin release from the NPs was evaluated using the Dialysis Method (DM) [29]. The Epi-NPs was investigated under physiological condition (PBS, pH 7.4) and simulated cancerous conditions (PBS, pH 5.5) *in vitro* over a 48 hours period. Freeze-dried drug-loaded NPs were suspended in water for self-assembly (final drug concentration 1 mg/mL) under gentle stirring for 30 min. As a control of epirubicin release rate from dialysis bag water solution of epirubicin was used (Epi concentration 1 mg/ml). Ten mL of the suspension was then placed inside the dialysis bag (Carl Roth, MWCO 12–14 kDa) in glass flasks that contained 100 ml phosphate-buffered saline (PBS), pH 7.4 and 5.5, as release media. The samples were kept at 37°C and were light-protected (laboratory shaker). Epirubicin release from Epi-NPs is higher in tumour simulating conditions due to lower pH. This is due to the pH-dependent bond between epirubicin and dextran nanoparticles that allows epirubicin release with lowering pH.

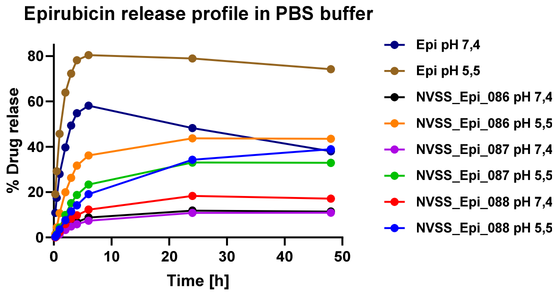

Figure S1. Epirubicin release profile determined by UV-Vis spectrophotometer.

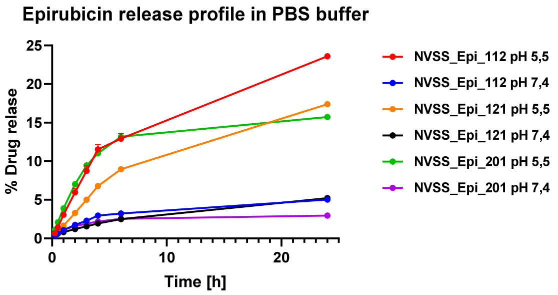

Figure S2. Epirubicin release profile determined by reversed-phase high-performance liquid chromatography (RP-HPLC).

3. Nanoparticles diameter and size distribution

The particle size and size distribution of NPs and POLEPI were determined with an LM 10 HS NanoSight instrument (Malvern Instruments Ltd.) with 405 nm laser scattering and Nanoparticles Tracking Analysis software. The measurements were replicated at least three times. The particle size of NPs is within range 107.3-137.2 nm and particle size of POLEPI is within range 88.6 -150.4 nm (**Error! Reference source not found.**).

Table S4. Empty NPs & POLEPI particle size

Sample / batch name	Particle size (nm)	Standard deviation (±nm)
**Empty nanoparticles (NPs)**		
NVSS111_carrier[a]	107.3	4.6
NVSS_carrier_219B	137.2	4.3
**POLEPI**		
NVSS112_Epi[a,c]	123.3	4.8
NVSS121_Epi	88.6	4.8
NVSS_Epi_201	117.5	5.8
NVSS_Epi_207	139.4	17.7
NVSS_Epi_209	150.4	10.6
NV_SS_EPi_219[b]	131.8	4.1

[a] lyophilized without cryoprotectant
[b] product without dialysis after synthesis
[c] zeta potential -3.13mV
